# Synthesis of Natural-Like Snow by Ultrasonic Nebulization of Water: Morphology and Raman Characterization

**DOI:** 10.3390/molecules25194458

**Published:** 2020-09-28

**Authors:** Ettore Maggiore, Matteo Tommasini, Paolo M. Ossi

**Affiliations:** 1 Dipartimento di Chimica, Materiali e Ingegneria Chimica “G. Natta”, Politecnico di Milano, Piazza Leonardo da Vinci 32, 20133 Milano, Italy; matteo.tommasini@polimi.it; 2 Dipartimento di Energia, Politecnico di Milano, Piazza Leonardo da Vinci 32, 20133 Milano, Italy; paolo.ossi@polimi.it

**Keywords:** artificial snow, laboratory snow, Raman spectroscopy, dendritic snow, OH-stretching, water nebulization

## Abstract

The current devices used to produce massive amounts of snow (i.e., snow machines) can be improved with concern to both the energy efficiency and the quality of snow. Here we investigate an alternative snow production method based on the ultrasonic nebulization of water and its subsequent condensation on the cold surfaces of a refrigerator. Inspection of the snow samples with a stereo optical microscope shows both dendritic and granular snow morphologies. The characterization of the samples by Raman spectroscopy shows a behavior consistent with that of a natural, low-density snow. Our results indicate that ultrasonic nebulization of water is an effective strategy for producing natural-like snow at the laboratory scale.

## 1. Introduction

Snow is an important material in nature. The high value of snow albedo makes it a fundamental control agent of the climate of our planet. The fraction of the Earth’s surface covered by snow determines the amount of solar radiation which on average is absorbed by the Earth [1]. In a different context, the availability of snow during the whole winter season is a prerequisite for tourism and the practice of winter sports on the mountains. Artificial snow produced by snow machines is used in the preparation of ski slopes and is obtained by the adiabatic cooling of high-pressure water sprays [2,3]. The cost of the produced snow is high: estimates range from ~1.5 €/m^3^ (including the installation and depreciation of the snow guns system and the energy cost) [4] to 5 €/m^3^ (including the cost for slope preparation and maintenance and personnel) [3]. The presently unavoidable fraction of liquid content (water) of artificial snow, as well as the snow quality, depend on the outdoor environmental conditions [5]. State of the art snow guns produce snow only below −5 °C (wet bulb temperature) but an acceptable solid to liquid ratio requires the external temperature to be lower than −6 °C [6]. Given the observed increase in average environmental temperatures of the last decades [7], the number of available days to produce artificial snow has reduced dramatically, in particular in places at medium-low altitude [7]. For this reason, it is desirable to develop alternative snowmaking techniques that can be employed in a wider temperature range and still guarantee good quality snow, while being environmentally sustainable.

From another perspective, the possibility of producing snow in a controlled way is fundamental in the laboratory for tribological studies (e.g., the sliding of ski bases [8], the mechanics of tires on snow [9], and the modeling of avalanches [10]). The sliding properties of a material on snow are affected by the snow grain morphology and size [11], which differ between natural and artificial snow [2]. For this reason, the availability of natural-like snow in the laboratory is also desirable.

A natural snow crystal grows inside a cloud from the frosting of the water vapor; its six-fold shape reflects the hexagonal symmetry of the crystalline structure of ice (I_h_) [12]. The principal morphologies that a snow crystal can display while growing have been classified in the landmark diagram obtained by Nakaya according to the environmental conditions of temperature and water vapor supersaturation [13]. The most common snowflakes are characterized by dendritic structures that grow symmetrically, starting from the vertexes of a single hexagonal crystal [14]. The fresh snow deposited on the ground undergoes metamorphism, i.e., the irreversible change in the shape of the crystals due to the redistribution of the water molecules through sublimation and frost cycles. The water vapor pressure gradients within a snow sample drives the metamorphism. Such vapor pressure gradients are generated by temperature gradients [15], differences in the radii of curvature (Kelvin effect) [16], or the presence of water in the liquid state [17,18,19,20].

Another phenomenon that occurs during metamorphism is the aggregation and subsequent sintering of individual crystals. This process causes a reduction of the specific surface area (SSA) of snow, which has a maximum value in freshly deposited snow and reduces as the dimension of the grains increases [21,22]. For this reason, to study and characterize individual snow crystals, it is necessary to sample them outdoors during a snowfall [23,24] or to synthesize them in the laboratory. There are several examples of laboratory devices designed to produce in a controlled way and to observe individual snow crystals [25,26]. The production of snow with morphology and physical properties like those of natural snow was recently demonstrated [27]. Water vapor is produced by heating liquid water and inducing ice condensation on nylon strings placed in a cold climatic chamber kept at about −20 °C. A snow production rate of ~200 g/h is reported in the case of the highest efficiency test. Here, we introduce a laboratory prototype able to produce quantities of snow of the order of 100 g/h, employing a simpler and cheaper experimental setup. Our device essentially consists of an ultrasonic nebulizer and a refrigeration cycle that makes the nebulizer nearly independent from external laboratory conditions. The produced snow that from now on will be defined as laboratory snow, has characteristics like those of natural snow. We recently used Raman spectroscopy to characterize different types of natural snow [28] and we observed that the features of the OH-stretching band correlate with the SSA of the analyzed sample. We compared these results to other samples with different SSA values and we observed consistent differences in the features of the OH-stretching region that became more relevant when approaching the melting temperature of ice. By taking advantage of this property we could distinguish different kinds of snow. Here we used Raman spectroscopy to characterize the snow samples produced by ultrasonic nebulization and we compared them with natural snow samples from Gressoney St. Jean (Lys Valley, Aosta, Italy).

## 2. Results

**Figure 1:**
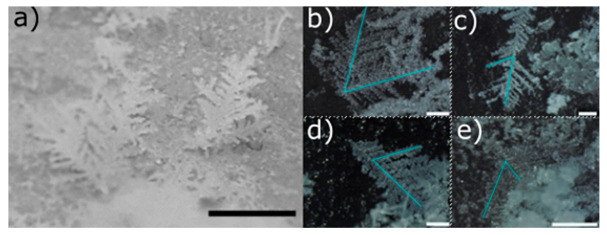
(**a**) Picture of a double and a single dendritic snow crystal grown on the internal surface of the freezer. Microscopic pictures of double (**b**) and single (**c**,**d**,**e**) dendritic snow crystals. Black and white bars are 1 cm and 500 µm long, respectively. The angle between the light blue lines displayed on the crystal branches is 60°.

**Figure 2:**
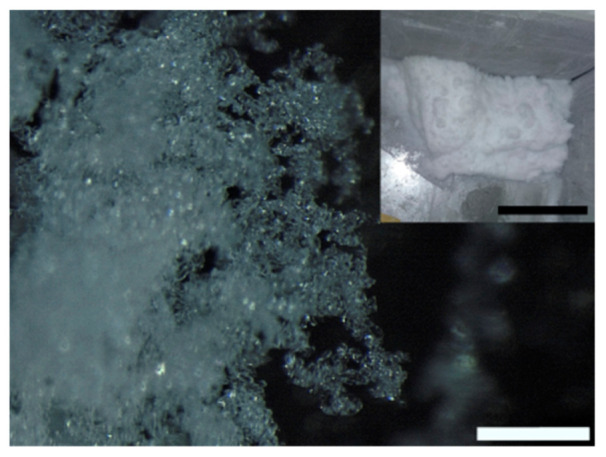
Microscopic picture of granular snow. Inset: snow accumulation produced after 3 h of deposition. Black and white bars correspond to 10 cm and 500 µm, respectively.

We display in Figure 1 and Figure 2 the different snow morphologies that we obtained after a 3 h production stage.

We discerned dendritic snow (Figure 1) from granular snow (Figure 2). Dendritic snow crystals formed in the upper part of the side walls of the freezer. The dendrites that we observed resembled the structures which develop at the corners of a dendritic snow crystal [13]. Most of the dendrites had only one principal branch which grew along the direction perpendicular to the side wall of the freezer. More rarely we found dendrites with two principal branches (Figure 1b) stemming from the same point on the surface, forming an angle of 60°. In all dendrites, we observed the same value of the angle (60°) between the principal and secondary branches (Figure 1b–e). We did not find crystals formed by more than two principal dendrites. The length of the dendrites varied according to the duration of the production cycle. Typically, in a 3 h production stage their size reached a value between 1 cm and 1.5 cm. Granular snow (Figure 2) formed on the bottom of the freezer. It is characterized by agglomerated rounded particles with an average dimension of ~50 µm.

The inset in Figure 2 shows a representative picture of the snow obtained after a 3 h production stage. We did not separate the two types of snow because the dendritic crystals are extremely fragile, and they break during snow collection. The density of the laboratory snow was 107 kg/m^3^ which is located in the range reported in the literature for fresh snows [29]. This value is close to that of the newly deposited natural snow that we measured in the mountains (Gressoney St. Jean, Aosta, Italy) during an early spring day (171 kg/m^3^).

We observed that the snow production rate of our device varied over time from 85 g/h during the first hour to 62.3 g/h during the second hour and 59.4 g/h during the third hour. This behavior can be explained by the worsening of the heat exchange between the cooler and the humid air in the cold chamber caused by the increased thickness of the progressively accumulated snow.

**Figure 3:**
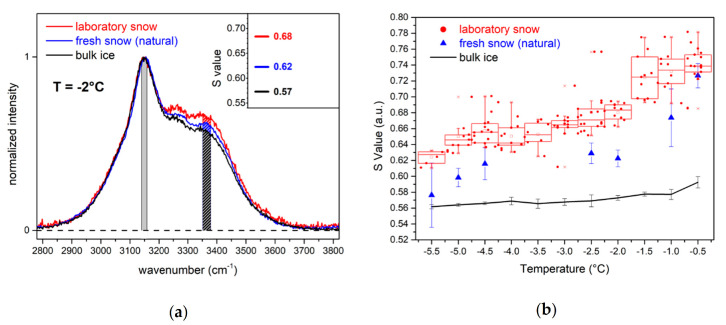
(**a**) Raman spectra taken at −2 °C across the OH-stretching band of bulk ice (black), fresh snow collected on field (blue), and laboratory snow (red). Shaded areas under the curves represent the integration intervals of Equation (1) for the three cases. The S values of the three spectra are reported in the inset. (**b**) S values of bulk ice (black line), fresh snow (blue triangles), and laboratory snow (red circles). The small red circles are the S values for all the laboratory snow spectra. The red box plot is obtained by grouping for a given T_0_ temperature the S values measured on laboratory snow over the temperature interval T_0_ − 0.25 °C < T < T_0_ + 0.25 °C.

We used Raman spectroscopy for a morphological characterization and comparison of the laboratory snow, the natural snow, and bulk ice. The technique has long been used for water and ice characterization based on the comparison of relative intensities of selected spectral features across the OH-stretching band [30,31]. More recently, the same analysis has involved different kinds of snow [28]. In Figure 3a the comparison of the normalized Raman spectra of natural and laboratory snow at the same temperature (−2 °C) displays different features.

The relative intensity of the laboratory snow spectrum in the region between 3200 cm^−1^ and 3500 cm^−1^ is higher than that of ice and natural snow. We calculated the S parameter (Equation (1)) for the spectra of the three samples, and we found that its value progressively increased from ice to natural snow and laboratory snow (inset in Figure 3a). The observation is valid for the entire considered temperature range (−5.5 °C < T < −0.5 °C) as shown in Figure 3b. For ice, the S value is almost constant, with a slight increase around −1 °C. Natural snow has a higher S value than ice. The trend increases with increasing temperature and is steeper when approaching the solid–liquid phase transition temperature (T > −1 °C). The S value of laboratory snow is the highest among the three samples and it is clearly distinguishable from that measured for natural snow down to −1 °C. The trend of the S value is linear between −5.5 °C and −4.5 °C, then decreases slightly at −4 °C and rapidly increases around −1.5 °C. Thereafter, it remains almost constant up to −0.5 °C.

## 3. Discussion

**Figure 4:**
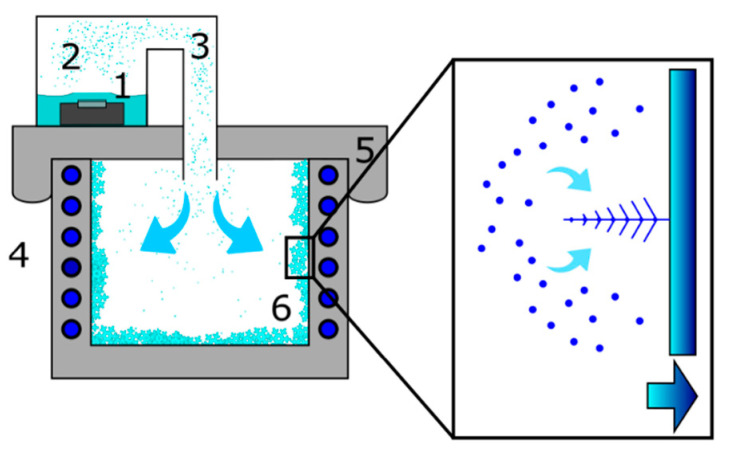
Schematization of the snow-making device. Ultrasonic nebulizer (1), liquid water supply (2), stream of nebulized water droplets (3), refrigerating fluid (4) that circulates around the freezer walls, Styrofoam plate (5), and snow deposit (6) that grows on the freezer internal surface. Magnification: mechanism of the generation of the snow dendrites on the metallic plates of the freezer. The blue arrow indicates the direction of the heat flow.

The droplets produced by the nebulizer enter the freezer chamber (Figure 4) and move downwards at a constant speed under gravity action while at the same time they are slowed down by the aerodynamic friction.

For the average diameter of the droplets (13.8 µm), the estimated maximum free fall speed is ~0.5 m/s. We used a model that considers the aerodynamic friction of spherical particles that propagate through ambient air [32]. In a short time, away from the freezer walls, the air becomes saturated with water vapor due to the evaporation from the surface of the droplets. Once this condition is reached, the droplets are in equilibrium with the environment and their size remains unchanged while moving. They reach the bottom wall of the freezer, where they immediately freeze on contact. This accumulation of the freezing droplets generates the observed granular morphology. The liquid droplets impinging on the cold surface may aggregate, thus the observed dimension of the solid particles (~50 µm) is larger than that of the original liquid droplets (~13.8 µm). The phenomenon does not occur on the side walls because the trajectory of the water droplets prevents a direct contact. The side walls of the freezer extract heat from the environment (Figure 4), therefore a supersaturated water vapor environment is locally created. In such conditions dendritic snow crystals nucleate and grow from supersaturated vapor. The morphology of the crystals grown on the side walls is determined by the temperature conditions of the surrounding air. In our case, we obtained dendritic crystals with an average air temperature of −15 °C, in agreement with the observations reported in the Nakaya morphology diagram [13].

The Raman spectrum of ice is sensitive to the hydrogen bonding network of water which changes during the solid-liquid transition [30]. We have recently shown the contribution from the ice surfaces in the Raman spectrum of natural snow, and we have related it to the water molecules at the surface which have a different hydrogen bonding network compared to the water molecules in the bulk [28]. We introduced an empirical parameter (S, see below) related to the spectral shape in the OH-stretching region that correlates with the SSA of snow [28]. For instance, we have shown that natural, fresh snow, characterized by crystals with a dimension of ~100 µm, has an S value higher than that of aged snow with crystals sized about 0.5 mm. The SSA of aged snow, due to the larger size of the crystals, is lower than that of fresh snow [21]. By carrying out a similar reasoning, we conclude that the larger S value of the laboratory snow compared to natural snow indicates that the SSA of laboratory snow is larger than that of natural snow. We could not perform the direct measurement of the SSA of the two samples, but the lower density value of the laboratory snow (107 kg/m^3^ < 171 kg/m^3^) and the smaller size of the constituting particles (50 µm < 100 µm) are indicative of a larger SSA, similar to literature studies [21].

## 4. Materials and Methods

In Figure 4 we display a schematic of our home-made device for laboratory snow production. The ultrasonic nebulizer (1) produces a stream of small water droplets (3) that flows inside the freezer. The snow crystals nucleate and grow on the metallic walls of the freezer (6). We used a 200 W commercial freezer with an internal surface of stainless-steel plates which remove the heat from the environment. We sealed the top opening of the freezer with a 2 cm thick Styrofoam plate on which we made a circular hole (diameter 5 cm). We adapted a polyethylene tube to the circular hole to connect the freezer with a water supply (2).

The ultrasonic nebulizer is a piezoelectric ceramic disk that resonates at 50 kHz and consumes 24 W. The throughput of the nebulized water inside the freezer is 200 mL/h. The droplets are ejected from the free surface of water which in our setup measured 320 cm^2^. This ensures a particle flux of about 5.3 × 10^5^ dropl./(cm^2^/s) assuming, for simplicity, that all droplets have the same average diameter of 13.8 µm ± 2.8 µm. Such droplet size was evaluated by depositing droplets on a flat glass sheet, then taking their size under the stereo microscope (see below for details). By considering the observed increase in the number of droplets per unit time on a surface of 100 µm^2^ we could estimate the particle flux, the order of magnitude of which was the same as the one mentioned above. To obtain an optimal nebulization, the water level above the piezoelectric resonator must be 2–3 cm high, therefore every 20 min we refilled the water holder. The measured temperature of the water bath during nebulizer operation was +35 °C.

After a snow production cycle (3 h) we removed the Styrofoam plate and collected the deposited snow from the five cold walls of the freezer (see inset in Figure 2). We stored the laboratory snow at −13 °C inside an environmentally sealed polyethylene container. The pictures of the snow samples were acquired with a smartphone camera and an Olympus SZX16 stereo microscope connected to an LC30 Olympus digital color camera. To keep an appropriate temperature of the snow samples during the microscope observation we built an in-house sample holder as follows. We filled a Styrofoam container with a solution of water and NaCl (8 M). At the container center we placed an aluminum heat sink with the smooth surface exposed to air. When the solution was frozen the temperature at the surface of the heat sink remained at −15 °C (melting temperature of the saline solution) until all of the solid melted (about 1 h at the external temperature of +25 °C). To acquire a picture with the stereo microscope, we carefully placed the snow sample on the aluminum surface, and we illuminated it with a blue LED lamp. Temperatures were recorded with a thermocouple thermometer (type K chromel-alumel) ensuring an accuracy of ±0.5 °C, a precision of ±0.1 °C (for temperatures −150 °C < T < +210 °C), and a refresh rate of 200 ms. We inserted the sensing component of the thermometer in the snow down to a depth of about 2 cm to take the sample temperature when performing Raman measurements. Weight measurements were acquired with a digital balance with a precision of ±1 g.

Raman spectra were taken with a portable Raman equipment BW&TEK Exemplar Plus model (wavelength range 532–680 nm; slit width 10 µm, diffraction grating 1800 lines mm^−1^; and resolution 2 cm^−1^). All spectra were acquired at a fixed laser power PL = 50 mW, with acquisition time of 15 s. We used a BAC102 Raman probe (BW&TEK) that operates in backscattering at a working distance of 5.4 mm (diameter of the laser spot, 85 µm). Raman spectra (RS) of the snow samples were taken by directly inserting down to ~1 cm beneath the snow surface the BAC102 probe, with its optical window protected by a thin transparent polyethylene film. Since the latter is located away from the focal plane of the probe lens the very weak contribution it carries to the RS can be safely subtracted from the recorded spectra.

We also produced a reference ice (I_h_) sample (1 × 1 × 1 cm^3^) by cooling down deionized water to −10 °C (conductivity at +25 °C, 0.055 μS cm^−1^). RS of ice were acquired by focusing the laser inside the bulk of the snow volume.

We collected RS of fresh natural snow at Gressoney St. Jean (Lys Valley, Aosta, Italy) during different campaigns of field measurements (2018–2019). RS were acquired between 12 and 24 h after a snow fall to avoid as much as possible metamorphism of the snow grains.

We collected RS of laboratory snow between −5.5 °C and −0.5 °C inside a temperature-controlled environment. We collected 10 spectra at different random places in the sample volume in the interval of temperatures (T − 0.5 °C) < T < (T + 0.5 °C). We considered eleven values of temperatures from −5.5 °C to −0.5 °C, separated from each other by 0.5 °C. We did not consider spectra acquired at temperatures higher than −0.5 °C. To collect the RS at each temperature we used a fresh laboratory snow sample. For each RS we calculated the S values following the same procedure described in [28]:

(1) $$S=\frac{\frac{\int_{3350}^{3380}I\left(\omega \right)d\omega }{\left(3380-3350\right){\mathrm{cm}}^{-1}}}{\frac{\int_{3140}^{3160}I\left(\omega \right)d\omega }{\left(3160-3140\right){\mathrm{cm}}^{-1}}}$$

where *I*(*ω*) is the intensity of the baseline corrected spectrum in the OH-stretching region (2800 cm^−1^–3800 cm^−1^) as a function of Raman shift.

## 5. Conclusions

We have shown the possibility of producing snow with properties similar to those of natural snow. We identified the mechanisms that produce the two snow morphologies we observed. In the upper region of the freezer chamber, dendritic snow grows from water vapor, while at the bottom of the freezer chamber, freezing of the liquid water droplets produced by the nebulizer results in the granular snow morphology.

The density of the laboratory snow is lower than that of fresh natural snow. Unlike the snow produced by snow guns, this snow production system does not have the drawback of the presence of a fraction of liquid water in the snow. Moreover, the low density of the laboratory snow allows for water to be saved while obtaining the same volume of snow, with obvious environmental advantages. In agreement with the small average size of the laboratory snow grains, the Raman spectra of the laboratory snow indicate a higher SSA than that of natural snow. With this snow production device, it may be possible to produce other kinds of snow by suitably modifying the environmental conditions inside the freezer chamber. The device discussed here is a simple system that allows for the production of enough snow for laboratory activity in a short time. The setup could be easily scaled up for other applications.
